# Potential drug-drug interactions in outpatient department of a tertiary care hospital in Pakistan: a cross-sectional study

**DOI:** 10.1186/s12913-018-3579-7

**Published:** 2018-10-10

**Authors:** Mohammad Ismail, Sidra Noor, Umme Harram, Inamul Haq, Iqbal Haider, Faiza Khadim, Qasim Khan, Zahid Ali, Tahir Muhammad, Muhammad Asif

**Affiliations:** 1 Department of Pharmacy, University of Peshawar, Peshawar, Khyber Pakhtunkhwa Pakistan; 2 Department of Medicine, Medical Teaching Institute, Khyber Teaching Hospital, Peshawar, Pakistan; 3 Department of Pharmacy, COMSATS Institute of Information Technology, Abbottabad, Pakistan

**Keywords:** Potential drug-drug interaction, Drug interaction, Outpatients, Drug related problem, Patient safety

## Abstract

**Background:**

Potential drug–drug interactions (pDDIs) are one of the preventable drug related problems having the risk of serious adverse events or therapeutic failure. In developing countries like Pakistan, this issue remains poorly addressed. The objective of this study was to explore prevalence of pDDIs in the Outpatient Department (OPD) of a tertiary care hospital in Pakistan. The secondary aim was to describe the levels of reported pDDIs and develop a list of widespread clinically relevant interactions.

**Methods:**

Prescriptions of 2400 OPD patients were analyzed for pDDIs through Micromedex Drug-Reax®. Prevalence, severity- and documentation-levels and widespread clinically relevant interactions were reported.

**Results:**

Of total 2400 prescriptions, pDDIs were present in 22.3%. Whereas, moderate- and major-pDDIs were found in 377 (15.7%) and 225 (9.4%), respectively. PDDIs were more prevalent in Medicine (9.2%) and Cardiology (2.6%) as compared with other OPD specialties. Total 942 pDDIs were identified, of which, the majority were either moderate- (61.9%) or major-pDDIs (32.1%). Some of the most common interactions were ibuprofen + levofloxacin (*n* = 50), ciprofloxacin + diclofenac (32), aspirin + atenolol (24), and diclofenac + levofloxacin (19). The potential adverse outcomes of widespread interactions were seizures, bleeding, QT-interval prolongation, arrhythmias, tendon rupture, hypoglycemia/hyperglycemia, serotonin syndrome, drug toxicity, and decreased therapeutic response.

**Conclusions:**

OPD patients were at risk to pDDIs, particularly to major- and moderate-pDDIs. Screening of prescriptions for pDDIs and monitoring of pharmacotherapy in terms of response and associated adverse drug events will contribute to patient safety.

**Electronic supplementary material:**

The online version of this article (10.1186/s12913-018-3579-7) contains supplementary material, which is available to authorized users.

## Background

Potential drug–drug interactions (pDDIs) are one of the preventable drug related problems having the risk of serious adverse events or therapeutic failure [1]. Their associated adverse drug reactions (ADRs) may lead to morbidity or mortality [2]. ADRs are responsible for nearly 5% of hospital admissions, of which, 0.25 to 25% are due to drug-drug interactions (DDIs) [1, 3–6]. Identification and management of DDIs are crucial for preventing the associated risk [7].

DDIs are highly prevalent in hospitalized patients and is well studied with respect to specific wards of the hospital [7–10], cause of hospitalization [1, 2], class of drugs [11, 12], and patient population [13–15]. Up to our knowledge, studies concerning the nature and prevalence of pDDIs in the outpatient department (OPD) of Pakistani hospitals remains unaddressed. Some studies from the developed countries have reported a prevalence rate of 28–83% for pDDIs in OPD [4, 14, 16–19]. These studies are limited by the nature of study settings, design, DDIs screening tool, and drug prescribing pattern.

Several factors are responsible for prevalence of pDDIs among outpatients. Healthcare professionals in developing countries, including Pakistan, face a number of challenges; they are overburdened [20], patients present with a wide range of illnesses [21], and lack of adequate preceding medical and medication records [20]. Moreover, the means to properly ascertain medication adherence, therapeutic outcome and ADRs are lacking [20, 21]. Taking into consideration all the above facts, it is vital to conduct study regarding pDDIs in OPD settings within Pakistan.

The primary objective of this study was to explore the prevalence of pDDIs in prescriptions for patients visiting OPD. The secondary objectives were to describe the levels (severity and documentation) for the identified pDDIs, and report the widespread clinically relevant interactions.

## Methods

### Study design and setting

This cross-sectional study was performed in the OPD of Khyber Teaching Hospital (KTH) Peshawar, Pakistan. KTH, a tertiary care hospital is one of the major hospitals of the provincial capital, providing healthcare services to the adjacent local population as well as referred patients from other districts.

Patients in OPD of KTH are checked/examined by physicians and prescriptions are written manually. The prescriptions are filled either by the in-house hospital pharmacy or an outside retail pharmacy based on patient preference. The hospital lacks a functional computerized information system for keeping record of the filled OPD prescriptions. Therefore, records are maintained manually in hospital pharmacy. Moreover, clinical pharmacy services and computer based drug interaction screening programs do not exist.

OPD in KTH comprise of various specialties such as medicine, pediatrics, psychiatry, ENT (ear, nose, and throat), dermatology, chest, cardiology, gynecology, surgical, eye, etc. Each OPD specialty provides distinct services to treat minor as well as complicated multi-organ disorders. Medicine-OPD offers an array of services, right from treating simple fevers, to complex multi-organ medical problems requiring consolidated attention and referral to proper specialty and hospital admission, if required. In Pediatric-OPD, pediatric patients are treated and referred. Dermatology specialty treats the patients with skin disorders, hair disorders like baldness, hair loss, and dandruff. Moreover, nail problems like abnormal nail growth or discoloration are also treated. ENT-OPD deals with the treatment of diseases of ear, nose, and throat. Similarly, Chest, Psychiatry, Cardiology, Gynecology, and Orthopedic-OPD provides specialty care for the treatment of respiratory, mental, cardiac, reproductive system, and joint disorders, respectively. While, Dentistry deals with treating the patients having problems related to teeth. The disease and drug prescribing pattern is different in all these OPD specialties.

### Administrative and ethical approval

Permission to access patients’ prescription records and ethical approval was obtained from the administration and ethical committee of the hospital, respectively.

### Data source

This study includes 2400 prescriptions of OPD patients, filled at the hospital pharmacy from August 2014 to February 2015. A prescription refers to an order for medication(s) issued by a licensed medical practitioner [22]. In inscription part of the prescription, all medicines are mentioned which are recommended by physicians for the patient. Therefore, a prescription contains a list of one of more prescribed medicines.

Prescriptions were excluded if these were incomplete with respect to relevant data needed for the study. Relevant data available in OPD prescription i.e., patient’s age, gender, clinical specialties, names and number of prescribed medications were collected retrospectively.

### Screening of prescriptions for pDDIs

Micromedex Drug-Reax® (Truven Health Analytics, Greenwood Village, Colorado, USA) [23] was used to evaluate prescribed drugs for the presence of pDDIs. According to description of this software, interactions were divided into various groups, based on severity- and documentation-levels as mentioned bellow [23]. Additionally, information regarding potential adverse outcomes of each DDIs is also provided.

Severity levels:

- Contraindicated: The drugs are contraindicated for concurrent use.
- Major: The interaction may be life threatening and/or require medical intervention to minimize or prevent adverse effects.
- Moderate: The interaction may result in exacerbation of the patient condition and/or require an alteration in therapy.
- Minor: The interaction would have limited clinical effects. Manifestations may include an increase in the frequency or severity of the side effects, but generally would not require a major alteration in therapy.

Documentation levels:

- Excellent: Controlled studies have clearly established the existence of the interaction.
- Good: Documentation strongly suggests the interaction exists, but well-controlled studies are lacking.
- Fair: Available documentation is poor, but pharmacological considerations lead clinicians to suspect the interaction exists; or documentation is good for a pharmacologically similar drug.

The overall prevalence of pDDIs as well as prevalence on the basis of severity levels (contraindicated, major, moderate and minor) was explored. Total interactions were classified on the basis of severity- and documentation-levels. All types- and major- pDDIs were stratified against patients’ characteristics and OPD specialties. Moreover, the list of most frequent pDDIs alongside their levels and potential adverse outcome were also reported.

### Statistical analysis

Quantitative variables, including patients’ age and numbers of prescribed medications are presented categorically as frequencies and percentages. Number of prescribed medications are presented as median and interquartile range (IQR) as well. Categorical variables, including gender, number of pDDIs per patient, severity- and documentation-levels of pDDIs are presented in the form of frequencies and percentages. SPSS-v23 was used for statistical analyses of the data.

## Results

Of total 2400 prescriptions, females represented 54.7%. Majority patients were aged ≤30 years (*n* = 1595; 66.5%) (Table 1). Four or more medicines were prescribed mostly (*n* = 1438; 60%). Moreover, majority of the prescriptions were from Medicine (28.6%), Pediatrics (25.4%) and Dermatology-OPD (9.8%) as shown in Table 1.

**Table 1:** General characteristics of study subjects (*n* = 2400)

Characteristics	Patients: *n* (%)
Gender	
Male	1088 (45.3)
Age (years)	
≤ 10	766 (31.9)
11–20	386 (16.1)
21–30	443 (18.5)
31–40	310 (12.9)
41–50	292 (12.2)
51–60	138 (5.8)
> 60	65 (2.7)
Prescribed medicines per patient	
≤ 3	962 (40.1)
4–6	1235 (51.5)
> 6	203 (8.5)
Drugs	
Median (IQR)	4 (3–5)
Clinical specialties	
Medicine	687 (28.6)
Pediatrics	610 (25.4)
Dermatology	235 (9.8)
Ear Nose and Throat	190 (7.9)
Surgical	132 (5.5)
Chest	124 (5.2)
Psychiatry	114 (4.8)
Cardiology	89 (3.7)
Gynecology	78 (3.3)
Orthopedic	73 (3)
Dentistry	39 (1.6)
Miscellaneous	29 (1.2)

- *IQR* Interquartile range

Of total 2400 prescriptions, at least one pDDI was present in 534 (22.3%). Whereas, at least one moderate- and major-pDDI were reported in 377 (15.7%) and 225 (9.4%), respectively. Lowest prevalence was recorded for contraindicated (28, 1.2%) and minor-pDDI (27, 1.1%). Moreover, 189 (7.9%) prescriptions were having two or more pDDIs simultaneously. Therefore, at a time pDDIs of different severity levels were present in many prescriptions.

**Fig. 1:**
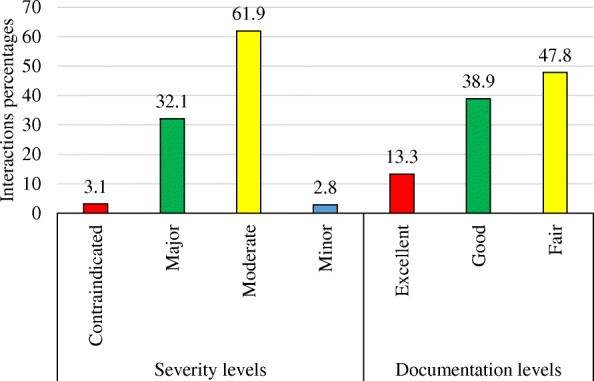
Severity and documentation levels of the identified potential drug-drug interactions

Figure 1 illustrates the severity- and documentation-levels of pDDIs. Total 942 interactions were identified. The majority were either moderate- (*n* = 583) or major-severity (302). Whereas, concerning scientific evidence, fair (*n* = 450) and good type of scientific evidence (366) were mostly observed.

Table 2 demonstrates exposure to all types- and major-pDDIs, stratified against patient’s characteristics. In females, the prevalence of pDDIs was greater as compared with males. The prevalence of all types-pDDIs was more among patients aged 21 to 30 years. While, major-pDDIs were highly prevalent among patients aged 10 years or less. Moreover, pDDIs were more frequent in patients to whom four to six medicines were prescribed (Table 2). Among OPD specialties, all types-pDDIs was more prevalent in Medicine- (*n* = 221), Cardiology- (62) and Psychiatry-OPD (58). Whereas, the prevalence of major-pDDIs was higher in Medicine- (*n* = 58), Pediatrics- (56) and Dermatology-OPD (19).

**Table 2:** Exposure to potential drug–drug interactions stratified with respect to patients’ characteristics

Characteristics	All types of interactions (*n* = 534)	Only major interactions (*n* = 225)
	*n* (%^a^)	*n* (%^a^)
Gender		
Male	230 (21.1)	101 (9.3)
Female	304 (23.2)	124 (9.5)
Age (Years)		
≤ 10	41 (5.4)	64 (8.4)
11–20	79 (20.5)	32 (8.3)
21–30	129 (29.1)	44 (9.9)
31–40	87 (28.1)	28 (9)
41–50	100 (34.2)	37 (12.7)
51–60	59 (42.8)	12 (8.7)
> 60	39 (60)	8 (12.3)
Prescribed medicines		
≤ 3	141 (14.7)	95 (9.9)
4–6	315 (25.5)	115 (9.3)
> 6	78 (38.4)	15 (7.4)
Clinical Specialties		
Medicine	221 (32.2)	58 (8.4)
Pediatrics	31 (5.1)	56 (9.2)
Dermatology	15 (6.4)	19 (8.1)
Ear Nose and Throat	36 (18.9)	17 (8.9)
Surgical	31 (23.5)	12 (9.1)
Chest	37 (29.8)	13 (10.5)
Psychiatry	58 (50.9)	11 (9.6)
Cardiology	62 (69.7)	12 (13.5)
Gynecology	19 (24.4)	13 (16.7)
Orthopedic	12 (16.4)	10 (13.7)
Dentistry	4 (10.3)	3 (7.7)
Miscellaneous	8 (27.6)	1 (3.4)

- ^a^Percentage within subsection of variable i.e., row wise percentage

Table 3 enlists widespread interactions, their levels and potential adverse outcomes. Ibuprofen and levofloxacin was the most prevalent interacting pair. Potential adverse outcomes of widespread interactions were seizures, bleeding, QT-interval prolongation, arrhythmias, gastrointestinal hemorrhage, antagonism of hypotensive effect, tendon rupture, hypoglycemia, hyperglycemia, serotonin-syndrome, drug toxicity, and reduction in therapeutic effectiveness (Table 3). An additional file enlists top 30 most frequently prescribed drugs (see Additional file 1).

**Table 3:** Most frequently identified interactions, their levels and potential adverse outcomes

Interacting Pair	Severity levels	Documentation levels	Patients: *n* (%^a^)	Potential adverse outcomes
Ibuprofen – Levofloxacin	Moderate	Fair	50 (2)	Seizures
Ciprofloxacin – Diclofenac	Moderate	Excellent	32 (1.3)	Increased ciprofloxacin plasma concentrations
Aspirin^b^ – Atenolol	Moderate	Good	24 (1)	Decreased antihypertensive effect
Diclofenac – Levofloxacin	Moderate	Fair	19 (0.8)	Seizures
Diclofenac – Metronidazole	Moderate	Fair	17 (0.7)	Increased exposure of diclofenac
Aspirin^c^ – Clopidogrel	Moderate	Fair	16 (0.7)	Bleeding
Ciprofloxacin – Metronidazole	Major	Fair	14 (0.6)	QT-interval prolongation
Amlodipine – Diclofenac	Moderate	Good	14 (0.6)	Gastrointestinal hemorrhage and/or antagonism of hypotensive effect
Levofloxacin – Prednisolone	Moderate	Excellent	13 (0.5)	Tendon rupture
Atorvastatin – Clopidogrel	Moderate	Excellent	13 (0.5)	Increased platelet reactivity
Aspirin^b^ – Glimepiride	Moderate	Good	13 (0.5)	Hypoglycemia
Glimepiride – Levofloxacin	Major	Fair	12 (0.5)	Hypoglycemia or hyperglycemia
Aspirin^c^ – Bisoprolol	Moderate	Good	12 (0.5)	Decreased antihypertensive effect
Levofloxacin – Thioridazine	Contraindicated	Fair	12 (0.5)	QT interval prolongation
Aminophylline – Levofloxacin	Major	Fair	12 (0.5)	Theophylline toxicity (nausea, vomiting, palpitations, seizures)

-^a^Percentage was calculated out of 2400, i.e., total number of patient’s prescriptions

-^b^Aspirin was prescribed as analgesic doses

-^c^Aspirin was prescribed as antiplatelet doses

## Discussion

This study identifies the prevalence and levels of pDDIs in OPD of a tertiary care hospital. The elderly patients (aged 51 and over) in our study were less (8.5% of total 2400) when compared to a recent study, in which elderly patients (aged 65 and over) were 29.4% of the total studied population [24]. This contradiction may be due to low literacy rate and health seeking behavior of elderly patients in Pakistan [25, 26]. In Pakistan, routine health care checkups are rare and patients usually present to hospital after severe or disabling complication of a disease, which often leads to hospital admission through the emergency department, thus bypassing the OPD. Most elderly patients are hospitalized in Pakistani settings [7].

Prevalence of pDDIs in our study is lower (22.3%) compared with that reported by similar studies from other countries (27.9–83.4%) [4, 14, 16–19], but in range with that reported by studies among hospitalized patients (19–70%) [7, 8, 27–29]. PDDIs were more prevalent in Medicine (9.2%) followed by Cardiology-OPD (2.6%). These findings are dissimilar to findings of a study conducted in Thailand—majority of pDDIs are observed in Psychiatry, followed by Medicine-OPD [19]. This inconsistency may be attributed to varied study population, study design, pattern of drug prescribing/utilization, disease trends, and type of DDIs screening tools. In developing countries, including Pakistan, OPD patients are at risk to DDIs and corresponding adverse events. Reasons may be overworked healthcare professionals, lack of proper treatment follow up and non-existing pDDIs screening facilities. Therefore, some specific strategies in the patient care process of OPD are suggested such as pDDIs screening system, adequate patient education and counselling, and regular follow-up.

Severity of pDDIs and their corresponding scientific evidence have a decisive role in the monitoring and management for adverse events related to interactions. We found moderate and major-pDDIs mostly, while, concerning scientific evidence, fair and good type. Our results are consistent with results of a study conducted among outpatients [17]. Similarly, studies among hospitalized patients also indicate similar results [7, 10, 27]. These findings reinforce the need of patient therapy monitoring through proper follow up for any adverse events due to concomitant administration of multiple drugs.

Monitoring and assessment for every single pDDI can be tedious, unproductive and further increase the burden on healthcare professionals. Moreover, limited number of pDDIs are of clinical importance due to negligible untoward effects. Every health care provider cannot differentiate pDDIs from ADRs, and take corrective measures accordingly [30]. Clinician’s knowledge about DDIs can decrease the likelihood of associated adverse outcome, able to provide better quality care, adjusts therapeutic regimen, and avoid associated medico-legal issues. Hence, clinical guidelines concerning the widespread pDDIs along with their potential adverse outcome and monitoring/management strategies should be developed. Most common interactions in our study were ibuprofen + levofloxacin, ciprofloxacin + diclofenac, aspirin + atenolol, and diclofenac + levofloxacin. While, other studies from the developed countries propose different DDIs such as anticoagulants + thyroid hormone, benzodiazepines + azole antifungals [18], macrolides antibiotics + HMG CoA reductase inhibitors, potassium sparing diuretics + potassium [31], isoniazid + rifampin, and digitalis glycosides + loop diuretics [19]. The reason for this inconsistency may be due to variable drugs prescribing/utilization pattern and DDIs screening system.

Following are the potential limitations of this study. We studied the pattern of pDDIs in different OPD specialties within a single setting. Although, a similar pattern is expected in other OPD settings, different findings are also possible due to variation in the nature of OPD settings. Therefore, multi-center studies are recommended. Moreover, the pDDIs were identified by using single drug interactions screening source (Micromedex Drug-Reax®), however, other sources are also available and differences exist among these drug interactions screening sources [32].

## Conclusion

A substantial prevalence of pDDIs has been observed in OPD (22.3%). Interactions of moderate-pDDIs were more common, however, major-pDDIs were also observed in considerable number. List of most frequently identified interactions will efficiently support the selective screening and monitoring of patients for pDDIs and associated negative consequences. To improve patient’s safety and outcomes of therapy, some strategies are essential such as software-based screening of pDDIs, patient education and counselling, and regular monitoring/follow-up.

## Additional file

Additional file 1: Top 30 most frequently prescribed drugs. (DOCX 14 kb)

## Abbreviations

ADRs: Adverse Drug Reactions
IQR: Interquartile Range
KTH: Khyber Teaching Hospital
OPD: Outpatient Department
pDDIs: Potential drug–drug interactions
