# Supplementary material for: Potential drug-drug interactions in outpatient department of a tertiary care hospital in Pakistan: a cross-sectional study
Source: BMC Health Serv Res. 2018 Oct 10;18:762. doi: 10.1186/s12913-018-3579-7 (PMC6186060; doi:10.1186/s12913-018-3579-7)
Supplement: Supplementary file 1 — Top 30 most frequently prescribed drugs. (DOCX 14 kb) [file 12913_2018_3579_MOESM1_ESM.docx]

**Additional Table 1: Top 30 most frequently prescribed drugs**

| **Prescribed drug** | **Frequency** |
| --- | --- |
| Paracetamol | 787 |
| Amoxicillin | 582 |
| Ibuprofen | 412 |
| Clavulanic acid | 398 |
| Cefixime | 275 |
| Multivitamin | 268 |
| Levofloxacin | 228 |
| Diclofenac | 223 |
| Metronidazole | 163 |
| Diphenhydramine | 157 |
| Cetirizine | 143 |
| Ciprofloxacin | 126 |
| Chlorpheniramine | 124 |
| Calcium | 124 |
| Clarithromycin | 119 |
| Ammonium chloride | 105 |
| Fexofenadine | 98 |
| Aminophylline | 94 |
| Pseudoephedrine | 94 |
| Aspirin | 90 |
| Pholcodine | 88 |
| Glimepiride | 79 |
| Sulphamethoxazole | 77 |
| Permethrin | 76 |
| Promethazine | 75 |
| Trimethoprim | 75 |
| Metformin | 74 |
| Mefenamic acid | 73 |
| Azithromycin | 71 |
| Folic acid | 68 |
